# A Low-Cost Method of Skin Swabbing for the Collection of DNA Samples from Small Laboratory Fish

**DOI:** 10.1089/zeb.2016.1348

**Published:** 2017-02-01

**Authors:** Carl Breacker, Iain Barber, William H.J. Norton, Jonathan R. McDearmid, Ceinwen A. Tilley

**Affiliations:** Department of Neuroscience, Psychology and Behaviour, College of Medicine, Biological Sciences and Psychology, University of Leicester, Leicester, United Kingdom.

**Keywords:** swabbing, fin clip, stickleback, zebrafish, genotyping, *Gasterosteus aculeatus*, *Danio rerio*, 3Rs

## Abstract

Fin clipping of live fish under anesthesia is widely used to collect samples for DNA extraction. An alternative, potentially less invasive, approach involves obtaining samples by swabbing the skin of nonanesthetized fish. However, this method has yet to be widely adopted for use in laboratory studies in the biological and biomedical sciences. Here, we compare DNA samples from zebrafish *Danio rerio* and three-spined sticklebacks *Gasterosteus aculeatus* collected via fin clipping and skin swabbing techniques, and test a range of DNA extraction methods, including commercially available kits and a lower-cost, in-house method. We verify the method for polymerase chain reaction analysis, and examine the potential risk of cross contamination between individual fish that are netted together. We show that swabbing, which may not require the use of anesthesia or analgesics, offers a reliable alternative to fin clipping. Further work is now required to determine the relative effects of fin clipping and swabbing on the stress responses and subsequent health of fish, and hence the potential of swabbing as a refinement to existing DNA sampling procedures.

## Introduction

The number of fish used in research has risen steadily over recent years, due to their ease of maintenance in the laboratory and similarities to other vertebrate species that make them useful alternatives for mammals in accordance with the principles of the “3Rs”—replacement, reduction, and refinement of animals in research.^1–5^ In 2015, 14% of all regulated animal procedures in Britain were undertaken on fish.^6^ The majority of these fish are small bodied, genome-sequenced model species that are suitable for biomedical and ecological study, such as the zebrafish *Danio rerio*^7^ and three-spined stickleback *Gasterosteus aculeatus.*^8^ The frequent use of these species in genetic studies means that DNA samples often need to be obtained *in vivo*, usually by fin clipping under nonterminal anesthesia^9^; however, the use of anesthetics may affect behavior and physiology.^10–12^ Furthermore, within the scientific community there is an increased awareness that fish may experience pain, stress, or lasting harm as a consequence of invasive procedures,^3,11^ driving the development of alternative methods.^5,11^

Swabbing skin mucus to collect DNA samples has been adopted for use in ecological studies of larger fish,^13–16^ and potentially represents a less invasive alternative to fin clipping. However, the lack of an established protocol for small-bodied model species means that it has yet to emerge as a routine protocol in the biomedical and biological sciences. In this study we validate a DNA swabbing protocol for zebrafish and three-spined sticklebacks, and determine the minimum body size necessary to obtain sufficient DNA for successful downstream polymerase chain reaction (PCR). In addition, we examine the potential for DNA cross contamination between individual fish when animals are in direct contact during netting. Finally, we compare DNA yields using different protocols that combine the swabbing technique with alternative DNA extraction methods. Our data suggest that the swab technique is a viable and reproducible alternative for the collection of DNA samples from small laboratory fish.

## Materials and Methods

### Fish supply and husbandry

Individual zebrafish (*D. rerio*) and three-spined sticklebacks (*G. aculeatus*) were selected from standard laboratory bred stocks maintained at the University of Leicester, UK, in accordance with local and national guidelines for animal welfare. The following zebrafish strains were used; AB wild-type, *casper*, *Tg(vmat2:GFP)*, *Tg(hb9:GFP)*, *Tg(glyt2:GFP).*^17–20^ Sticklebacks were bred from wild parents caught at Carsington Water, Derbyshire, UK (53° 3′32′′N, 1° 37′42′′W) using standard IVF techniques.^21^

### Swabbing and fin clipping procedures

**FIG. 1.**
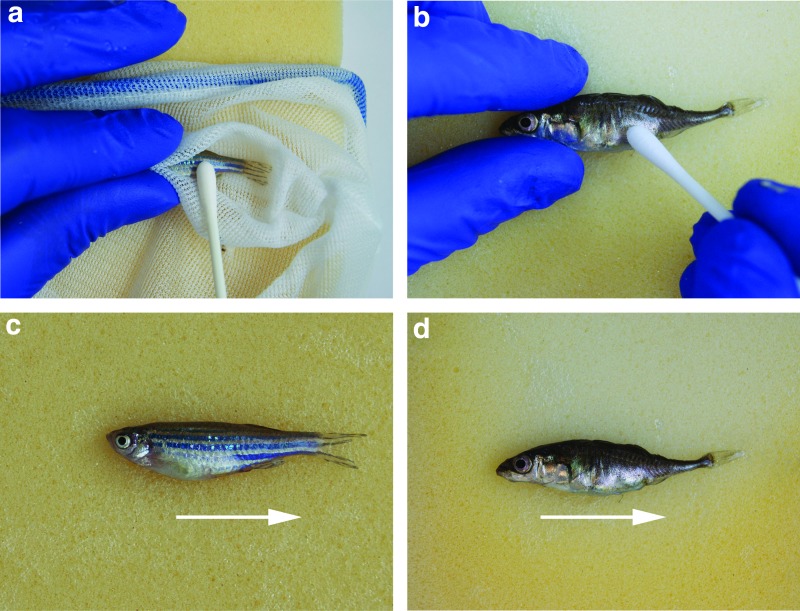
**(a)** Restraint and swabbing of a live zebrafish held within an aquarium net. **(b)** Restraint and swabbing of a live stickleback directly on a sponge**. (c)** *Arrow* indicates direction of swabbing on zebrafish. **(d)** *Arrow* indicates direction of swabbing on stickleback. Color images available online at www.liebertpub.com/zeb

Ten nonanesthetized adult fish of both species, all exceeding 40 mm standard length (SL), were either restrained in a groove cut into a wetted sponge, or held in an aquarium net on top of a flat wetted sponge, and secured with thumb and forefinger for zebrafish (Fig. 1a). Sticklebacks were placed directly onto the sponge and secured with thumb and forefinger (Fig. 1b). Skin mucus samples were collected by gently stroking with the tip of a sterile rayon-tipped swab (Code No. 11369633. Fisher Scientific) five times along the flank of each fish, from the operculum to the caudal fin (Fig. 1c, d). While the direction of swabbing is especially important in zebrafish, which possess scales, it may be less critical in sticklebacks, which lack scales but possess a variable number of bony lateral plates.^22^

For comparison, DNA was also obtained from an additional 10 adult fish from both species, using standard fin clipping procedures. Fin clips were taken from zebrafish held under nonterminal anesthesia (168 mg/L ethyl 3-aminobenzoate methanesulfonate [MS-222] buffered to pH 7.2 with sodium bicarbonate) and from sticklebacks under terminal euthanasia by overdose of benzocaine anesthetic (stock solution 10 g/L in 70% EtOH, diluted one in four in dH_2_O). Fins were clipped using a sterile surgical blade, so that no greater than half the caudal fin was removed.

In addition, we also subjected 10 *Tg(vmat2:GFP)* zebrafish and 10 adult sticklebacks to both procedures, with swabbing performed before anesthetizing and fin clipping.

### Assessment of detrimental effects

Fish used in swabbing and/or fin-clipping procedures were housed in aquaria connected to filtered, recirculating systems and monitored for 4 weeks postsampling to check for detrimental side effects (behavioral alterations and infection/swelling at the site of DNA collection).

### DNA extraction procedures

DNA was extracted from swabs using one of three commercially available kits, or an in-house method modified from an isopropanol precipitation protocol described by Sambrook and Russell.^23^ For each of the commercial kits, the method of extraction followed manufacturer's instructions. For the in-house method, the swab was first placed into a 1.5 mL microcentrifuge tube containing 400 μL DNA extraction buffer (200 mM TRIS pH 7.5, 25 mM ethylenediaminetetraaceticacid pH 8.0, 250 mM NaCl, and 0.5% w/v sodium dodecyl sulfate) warmed to 55°C, and incubated at room temperature for 15 min. The swab was then removed and 400 μL of chilled isopropanol was added to the DNA solution and mixed using a pipette. The DNA solution was then chilled at −80°C for 10 min. The solution was centrifuged for 10 min at 13,000 rpm, the supernatant decanted, and the remaining pellet washed with 190 μL 70% EtOH. After a further centrifugation step (2 min at 13,000 rpm) the DNA pellet was air dried and resuspended in 30 μL ddH_2_O. A similar protocol was followed for fin clip extractions, except in the initial step 15 μL of 20 mg/mL proteinase K was added to the DNA extraction buffer, followed by incubation at 57°C for 30 min before proceeding as above.

The concentration and purity of DNA from swabs taken from five different fish per extraction method were quantified using a NanoDrop 1000 spectrophotometer (LabTech International). Full laboratory methods used for swabbing fish and the in-house method can be found in supplementary data; supplementary data is available online at www.liebertpub.com/zeb).

### Effect of body size and strain on DNA sampling

To examine how fish body size affected the DNA yield recovered using the swabbing procedure, fish ranging in SL from 20 to 55 mm were swabbed. For zebrafish, results were validated across a number of different strains [AB wild-types (WTs), *casper*, *Tg(vmat2: GFP)*, *Tg(hb9:GFP)*, and *Tg(glyt2:GFP)*]. Eight fish were swabbed for each size and strain investigated.

### Potential for cross contamination

To assess the potential for cross contamination by mucus transfer between fish, adult AB WT zebrafish (*n* = 10) and adult *Tg(vmat2:GFP)* zebrafish (*n* = 10), both >40 mm SL, were housed together in a 3-L aquarium for ∼16 h. *Tg(vmat2: GFP)* carry a green fluorescent protein (GFP) transgene that can be amplified by PCR. To mimic standard husbandry procedures, immediately before swabbing all fish were scooped into a single net, and held out of the water for a few seconds, ensuring that fish came into direct contact with one another. After returning the fish to the water, AB WT fish were caught and swabbed. We selected long-finned *Tg(vmat2:GFP)* zebrafish that are visually distinguishable from AB WTs. The identity of individual swabbed fish was therefore known, allowing any contamination to be identified by attempting to amplify the GFP sequence from the nontransgenic AB wild-type fish. Following DNA extraction, PCRs were carried out using both *mitfa* and GFP primers. *mitfa* primers were used as positive controls for both strains of fish and to test the GFP primers DNA samples from identified GFP mutant fish were also used in the PCR reaction.

### PCR conditions

DNA samples obtained from three-spined sticklebacks in the study were used to genotype the donor fish, using a sex-linked molecular marker, isocitrate dehydrogenase. We used primers modified from those described by Peichel *et al.*^24^: (STKSEX forward primer 5′ GGGACGAGCAAGATTTATTGG 3′; STKSEX reverse primer 5′ TATAGTTAGCCAGGAGATGG 3′). Females produce a single band of ∼300 bp, while males produce two products of 270 and 300 bp due to a small deletion. Ten microliter PCR reactions were set up (5 μL Red Taq master mix (Sigma-Aldrich), 0.5 μL of the forward and reverse primer, 3 μL of DNA template, and 1 μL ddH_2_O). The reaction conditions of the PCR were 94°C for 5 min, followed by 40 cycles of 95°C for 30 s, 56°C for 30 s, 72°C for 30 s, with a final extension of 72°C for 10 min. PCR products were visualized on a 5% agarose gel.

For zebrafish, PCR conditions for AB WT and GFP identification were set up. This used primers designed against the genes coding for Mitfa (for WT) and GFP for *Tg(vmat2:GFP)*. The primers used are as follows: *mitfa* forward primer 5′ GCCAACTAAATTTCATGAACC 3′; reverse primer 5′ AAATCAACTAATTGTTTACACG 3′as described by Lister *et al.*^25^ and GFP forward 5′ TCGAGCTGGACGGCGACGT 3′; reverse 5′ GGTGCTCAGGTAGTGGTTGTC 3′. Ten microliter reactions were set up (5 μL Red Taq master mix [Sigma-Aldrich], 0.5 μL of the forward and reverse primer, 3 μL of DNA template, and 1 μL ddH_2_O). The reaction conditions of the PCR were 94°C for 2 min, followed by 35 cycles of 94°C for 30 s, 60°C for 30 s, 72°C for 1 min, with a final extension of 72°C for 10 min. Products were visualized on a 2% agarose gel.

## Results

### Qualitative assessment of alternative swabbing procedures

We found the most effective method for restraining fish during skin swabbing was to hold netted fish on a flat, wetted sponge. Using the grooved sponge was time consuming; the fish had to be positioned carefully, making it harder to access the flank area and prolonging the time spent out of water. Gently covering the eyes within the net also appeared to reduce fish movements during the procedure, allowing swabs to be taken more efficiently. During swabbing, each fish was out of the water for no more than 15 s when using the net method, compared to 30 s when trying to use the grooved sponge. In our hands, we found the time taken to conduct a swabbing procedure was considerably shorter than the total time taken to obtain a fin clip, when allowing for associated anesthesia and recovery periods. After 4 weeks, none of the fish (swabbed and/or fin-clipped) showed any sign of fungal infections, bacterial infection, or stress-related behavioral symptoms.

### Efficacy of swabbing technique for DNA sampling

The concentration and purity of DNA samples achieved using the various extraction methods are shown in Table 1. In our experiment the DNA yields achieved using the commercial kits varied considerably, with the Zymo Research Quick-gDNA MiniPrep kit performing best. The Bioline ISOLATE II Genomic DNA Kit achieved the next highest DNA yield, but also had the longest protocol with several additional steps compared to other commercial kits. A third kit (Kit “X”) gave lower recovery levels and also had the highest carry-over of ethanol with the extracted DNA as determined by low purity (260/230) ratios. In comparison, the in-house protocol gave yields and purities of DNA that were comparable to the best performing kits, and also worked well in the downstream PCR. We therefore used the in-house extraction method for all subsequent experiments.

**Table 1.** Concentration and Purity Estimates of Zebrafish DNA Recovered from Skin Swab Samples Using a Range of Commercially Available Kits and the Described In-House Extraction Methods Adapted from Sambrook and Russell^23^

*Extraction method*	*DNA concentration (ng/μL) (mean ±1 SD)*	*DNA purity*^a^*(260 nm:280 nm absorbance ratio) (mean ±1 SD)*	*DNA purity*^b^*(260 nm:230 nm absorbance ratio) (mean ±1 SD)*
ISOLATE II Genomic DNA Kit (Bioline cat. # BIO-52066)	24.34 ± 6.59	2.01 ± 0.11	1.38 ± 0.34
Quick-gDNA MiniPrep kit (Zymo Research cat. # D3024)	55.88 ± 11.71	1.95 ± 0.06	1.88 ± 0.11
Kit “X”	2.84 ± 2.16	1.80 ± 0.09	0.42 ± 0.14
In-house method	34.84 ± 6.19	1.99 ± 0.09	1.62 ± 0.36

*N* = 5 fish (SL >40 mm) per method.

^a^ 260:280 ratio is used to assess the purity of DNA; a ratio of ∼1.8 is generally accepted as “pure” for DNA.

^b^ 260:230 ratio is used as a secondary measure of nucleic acid purity. Lower values can indicate the presence of contaminants that absorb at 230 nm (including ethanol).

SL, standard length; SD, standard deviation.

### Comparison of DNA extraction from swabs and fin clips

**FIG. 2.**
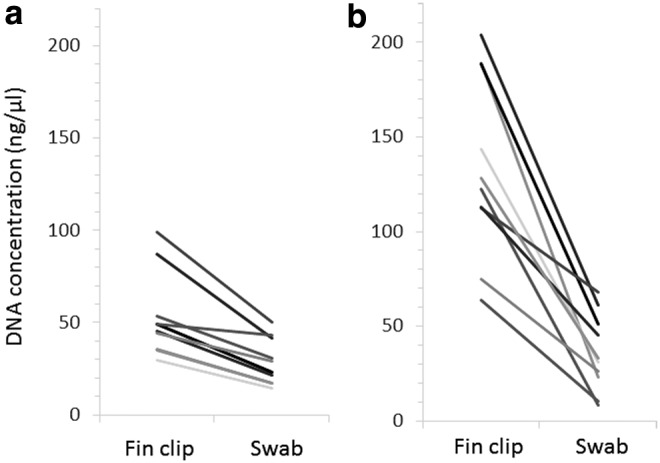
Concentration of DNA following sample extractions from fin clips and swabs from **(a)** zebrafish and **(b)** three-spined sticklebacks. In each case, fin clips and swabs were taken from 10 individual adult fish, represented by the separate lines on each figure.

**FIG. 3.**
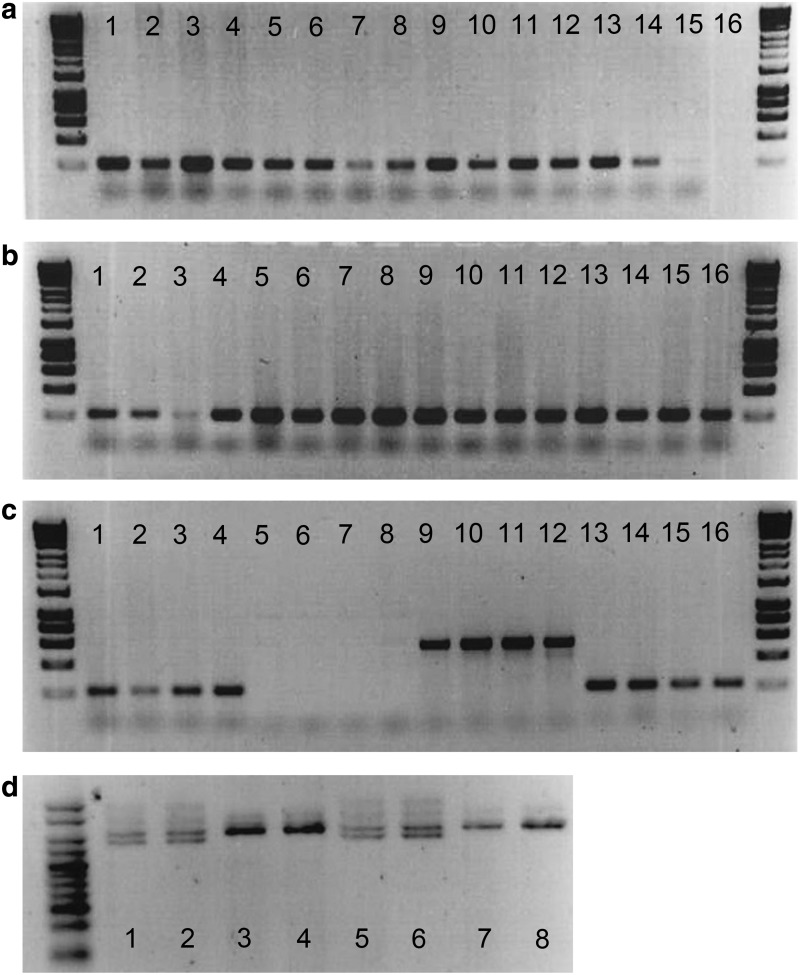
PCR results from DNA samples. **(a)** Zebrafish fin clips, swabs and size range; 1–3 = fin clips; 4–6 = swabs; 7–9 = swabs from 20 mm SL; 10–12 = swabs from 35 mm SL; 13–15 = swab from 50 mm SL; 16 = blank. **(b)** Zebrafish strains; 1–4 = Casper swab; 5–8 = HB9 swab; 9–12 = Glyt2 swab; 13–16 = Vmat. **(c)** Zebrafish cross contamination test; 1–4 = WT DNA with Mitfa primers; 5–8 = WT DNA with GFP primers; 9–12 = Vmat DNA with GFP primers; 5–8 = Vmat DNA with Mitfa primers. **(d)** Stickleback fin clips, swabs and size; 1–2 = 50 mm SL male swab; 3–4 = 50 mm SL female swab; 5–6 = 20 mm SL male swab; 7–8 = 20 mm SL female swab. SL, standard length; GFP, green fluorescent protein; WT, wild-type.

Comparing swabs and fin clips taken from the same fish revealed that fin clips produced higher DNA yields (Fig. 2). However, the swabbing method consistently generated sufficient DNA for successful PCR. When the amount of DNA used from fin clips and swabs was normalized across PCRs, very little difference was observed in the final amplified products, as evidenced by gel images (Fig. 3).

### Fish size and strain

Swabbing fish of different sizes (20 and 30 mm SL) produced comparable results to the largest fish (55 mm SL); however, we do not recommended the technique for fish smaller than 20 mm due to handling difficulty and the potential for injury given the size of the swab tip. We therefore suggest that this technique may be safely used on individuals smaller than those reported in recent studies (on African cichlids *Neolampologus pulcher*^15^), where the minimum sized trialled has been ∼40 mm. Different strains of zebrafish produced similar DNA yields and PCR results, indicating that genotype does not alter the results obtained (Fig. 3). Using the sex marker primers in sticklebacks, males and females could be identified even before morphological characteristics could be used to identify them (Fig. 3).

### Potential for cross contamination

With the mixed strain fish held at high density, no evidence for cross contamination was found. The *mitfa* primers gave rise to PCR products from both the wild-type fish and the *Tg(vmat2:GFP)* fish, indicating that the DNA extractions were adequate for downstream applications The gene coding for GFP was amplified from DNA obtained from *Tg(vmat2:GFP)* fish, while no amplification occurred from DNA extracted from wild-type fish. (Fig. 3).

## Discussion

In this article we provide a detailed protocol for using skin swabbing for the collection of DNA samples from small bodied, laboratory fish, and validate the technique for use with zebrafish and sticklebacks. We also document a low-cost method for the recovery of DNA from swab samples. Our results suggest that skin swabbing, combined with the low-cost recovery method, can yield DNA concentrations and purities that are comparable to those obtained from fin clips and commercially available DNA extraction kits. We therefore suggest that skin swabbing may provide a viable alternative to fin clipping as a method for DNA sampling of fish used in research.

While previous studies have used skin swabs to collect DNA samples from larger bodied fish species (e.g., bluegill sunfish *Lepomis macrochirus*,^13^ Atlantic cod *Gadus morhua*,^14^ African cichlids,^15^ and the Nile tilapia *Oreochromis niloticus*^16^) the technique is not routinely used in laboratory studies, which instead use fin clips from the caudal fins of live anesthetized fish.^9^ However, there are a number of reasons why fin clipping may not be an ideal technique for use in laboratory studies. First, there is evidence that fin clipping can generate behavioral and other side effects that may affect the outcome of experimental studies, including the potential for secondary infections and an elevated nonspecific immune response.^26^ Second, since some fish also use fins in communication or in reproductive behaviors, and for swimming^27–29^ fin clipping could influence behavioral interactions, reproductive success, or locomotion. Third, since fin clipping also requires fish to be held under nonterminal anesthesia—which can increase levels of cortisol^30–32^—there may be unintended consequences for studies of behavior or other endpoints that are influenced by stress responses, such as immune function. Fin clipping may also raise welfare concerns^10–12^ and, as it is classified as a regulated procedure in many countries, can often only be carried out in designated establishments by appropriately licenced researchers.

Skin swabbing has the potential to offer a less invasive method for obtaining DNA samples, though further research is now needed to determine its effects on the stress reponse and subsequent health of fish. The technique does not require the fish to be anesthetized, negating issues around the use of anesthetics. We did not investigate cortisol levels in our study; however, the lack of anesthesia and the removal from water for <15 s suggests a minimal effect.^33^ While we recorded no mortality, secondary infections, or aberrant behavior among the swabbed fish over a 28 days post-treatment period, more detailed studies investigating a wider range of responses would be needed to determine significant benefit of the swabbing approach over fin clipping. We suggest that comparative histological, microbiological, and immunological studies of fish subjected to swabbing versus fin clipping should now be undertaken to determine any 3Rs benefits.

Isolating DNA from zebrafish tissue samples is frequently undertaken using commercially available kits, or by the “hotshot” method, which involves incubating the sample in NaOH and neutralising using a Tris buffer solution.^34,35^ We attempted DNA isolation from swab samples using a range of commercially available kits alongside an in-house, low-cost method, and found the in-house method to perform at an equivalent level to two of the kits, and out-perform one of them. Further work would be needed to confirm whether the “hot-shot” method would also be suitabile for extracting DNA from swab samples, as this was not explicitly tested.

Because laboratory fish are typically housed in groups at high density^15^ and are frequently subjected to husbandry practices that bring them into direct skin contact (e.g., during netting), one possible problem with the use of skin samples is the potential for cross contamination between individuals resulting from mucus transfer. We tested this by screening skin swab samples taken from known-genotype fish for DNA markers possessed by co-netted tank-mates. We found no evidence of cross contamination among our samples, and in this study fish were housed at higher densities than those reported by others,^15^ confirming their findings that this is a robust technique for identifying individuals. Stocking densities used in this study were higher than the presently published guidelines of five fish per liter,^36,37^ suggesting that recommended housing densities and standard netting practices pose no major risk for the technique. Therefore, the chances of fish transferring mucus and DNA is potentially lower in standard facility set ups, meaning the suitability of this procedure over live fin clipping is high. Our results also suggest that the small size of laboratory fish poses no major problem, with useful DNA concentrations being recovered from fish as small as 20 mm SL. This is considerably smaller than previously reported in recent studies, where the minimum sized fish tested had been ∼40 mm^14,15^

In summary, skin swabbing appears to provide a reliable and efficient method for sampling DNA of small-bodied laboratory fish, which are among the most commonly used vertebrate models in modern laboratory research. While we have verified this technique for just two species—the zebrafish and the three-spined stickleback—we would suggest that it has wide applications and should be useful for a wide range of fish used in laboratory research, though further studies are now needed to confirm the extent of any potential welfare benefits.

## Ethical Statement

All work was undertaken under the authority of a UK Home Office project licence, in accordance with local and national regulations.

## Supplementary Material

Supplemental data
