# Supplementary material for: A Low-Cost Method of Skin Swabbing for the Collection of DNA Samples from Small Laboratory Fish
Source: Zebrafish. 2017 Feb 1;14(1):35–41. doi: 10.1089/zeb.2016.1348 (PMC5312459; doi:10.1089/zeb.2016.1348)
Supplement: Supplemental data [file Supp_Data.pdf]

Supplementary Data

Protocol for Fish Swabbing

1. Set up holding tanks for the total number of fish to be swabbed.
2. Label each swab tube with the ID number for the fish to be used.
3. Undo the top of the swab tube so the swab is easily removed from the tube when the fish has been netted.
4. Using a small sponge, wet the sponge with system water to keep the fish wet while swabbing.
5. Quickly net the fish you want to identify and place on the sponge, gently holding the fish around its head to keep it still.
6. With a clean swab for each fish, quickly but gently rub the swab along the fish's side, from head to tail, 5–10 times. Very little pressure is required—simply resting the swab on the fish and moving it along should pick up enough mucus to get a successful DNA sample.
7. Once the swab has been collected return it to its tube and put the fish in its holding tank until identification has been made.
8. Rinse the net used before netting the next fish and repeating steps 4–7 until the sample of fish required has been completed.
9. Once all the fish needed have been swabbed return to the lab to carry out the DNA extraction. This should be done as quickly as possible after the swabs have been taken.

DNA Extraction Solutions

Stock solutions:
1 M TRIS pH 7.5
0.5 M ethylenediaminetetraaceticacid (EDTA)
2 M NaCl
10% sodium dodecyl sulfate (SDS)

DNA extraction buffer (100 mL):
20 mL 1 M TRIS pH 7.5
5 mL 0.5 M EDTA
12.5 mL 2 M NaCl
57.5 mL dH_2O

Autoclave:
Once autoclaved add 5 mL 10% SDS (NB: do not autoclave once SDS has been added)

Isopropanol:
This is propan-2-ol, purchased in 2.5 L batches and stored in a fume cupboards.

Protocol for DNA Extraction from Swabs

Step 1:

- Prewarm DNA extraction buffer by incubating at 55°C (red oven in 331).
- Into a 1.5 mL eppi tube pipette 400 μL DNA extraction buffer. Place swab into this and cut off stem to top of tube so the lid can be closed.
- Vortex for 10–15 s.
- Incubate at RT °C for 15 min.

Step 2:

- Prechill isopropanol in –20°C.
- Remove swab and squeeze on side of tube to retain as much solution as possible.
- Pipette 400 μL of isopropanol into supernatant.
- Mix tube 3–5 times.
- Put into –80°C freezer for at least 10 min but can be overnight.

Step 4:

- Take out of –80°C and allow to defrost.
- Once defrosted centrifuge for 10 min at full speed.
- Dry the pellet by gently pouring solution away onto tissue.

Step 5:

- Add 190 μL 70% EtOH gently flick tube.
- Centrifuge for 2 min at full speed.
- Dry pellet with P200 set at 200 μL and heated in heat block set at 55°C for 5–10 min to fully dry pellet.

Step 6:
Pipette into tube 30 μL ddH_2O to resuspend DNA. Incubate in dry heat block at 65°C for 5–10 min. DNA is ready for PCR (use at least 3μL of DNA in PCR).
